# Apolipoprotein M T-778C polymorphism is associated with serum lipid levels and the risk of coronary artery disease in the Chinese population: a meta-analysis

**DOI:** 10.1186/1476-511X-12-135

**Published:** 2013-09-16

**Authors:** Zhi Zhang, Guang Chu, Rui-Xing Yin

**Affiliations:** 1 Department of Cardiology, Institute of Cardiovascular Diseases, The First Affiliated Hospital, Guangxi Medical University, Nanning 530021, Guangxi, People’s Republic of China; 2 Department of Cardiology, Shanghai First People’s Hospital, Shanghai Jiao Tong University School of Medicine, Shanghai 200080, People’s Republic of China

## Abstract

**Background:**

The apolipoprotein M (APOM) T-778C gene polymorphism has been associated with serum lipid levels and the risk of coronary artery disease (CAD), but the results are inconclusive. The purpose of this meta-analysis was to detect the association between the *APOM* T-778C polymorphism and serum lipid levels and the risk of CAD in the Chinese population.

**Methods:**

Databases of MEDLINE, EMBASE, the Cochrane Library and CNKI were systematically searched. Data were extracted using standardized methods. The association was assessed by mean difference (MD) with 95% confidence intervals (CI) or odds ratio (OR) with 95% CI.

**Results:**

Ten studies with 4,413 patients were included in this meta-analysis. Pooled effects indicated that CT+CC group had higher levels of total cholesterol (TC) (MD:-0.36, 95% CI: -0.53 – -0.19, *P* < 0.0001) and low-density lipoprotein cholesterol (LDL-C) (MD: -0.08, 95% CI: -0.16 – -0.01, *P* = 0.03) than TT group. There was no difference in the levels of triglyceride (MD: 0.06, 95% CI: -0.04 – 0.15, *P* = 0.22) and high-density lipoprotein cholesterol (MD: 0.00, 95% CI: -0.03–0.03, *P* = 0.93) between TT and CT+CC groups. Pooled effects showed that CAD group had higher CT+CC genotype frequency than control group (OR: 1.97, 95% CI: 1.62–2.39, *P* < 0.00001; heterogeneity test *x*^2^ = 2.96, *P* = 0.71, *I*^2^ = 0%).

**Conclusions:**

The results of the current meta-analysis show that the CT+CC group has higher levels of TC and LDL-C than the TT group. Moreover, there is also a prominent association between *APOM* T-778C polymorphism and the risk of CAD in the Chinese population, the CT+CC genotype is associated with increased risk of CAD.

## Introduction

Coronary artery disease (CAD) continues to be the leading cause of morbidity and mortality among adults nowadays [1]. Many studies have proved that serum lipid concentrations are strongly correlated to the risk of CAD, such as a low concentration of high-density lipoprotein cholesterol (HDL-C) [2,3], and high levels of total cholesterol (TC) [4], triglyceride (TG) [5], low-density lipoprotein cholesterol (LDL-C) [6], and apolipoprotein (Apo) B [7,8]. However, it is widely accepted that dyslipidemia and CAD are caused by multiple environmental and genetic factors and their interactions [9].

Association of different genes and CAD and/or dyslipidemia has been widely examined. Recently, apolipoprotein M (APOM) gene has been reported relevant to CAD and dyslipidemia [10]. APOM gene is located in p21.31 on human chromosome 6 (chromosome 17, in mouse). Human APOM cDNA (734 base pairs) encodes 188-amino acid residue-long protein [11]. APOM is mainly associated with HDL-C and lesser extent with TG-rich lipoproteins, ApoB-containing lipoproteins, low and very low-density lipoprotein (VLDL). It has been also demonstrated that APOM plays an important role in reverse cholesterol transport [12]. It is exclusively expressed in liver and in kidney [13].

Recent studies have showed that the *APOM* T-778C (rs805296) is valid in the Han Chinese and associated with serum lipid levels and the risk of CAD, but the results are inconclusive [14-23]. It may be partially due to the fact that the APOM gene was a minor gene for risk of dyslipidemia and/or the relatively small sample size in each of the published studies. Therefore, we performed a meta-analysis of the published studies to derive a more precise estimation of the association.

## Materials and methods

### Search strategy

All studies that reported the association between the *APOM* T-778C polymorphism and serum lipid levels and/or the risk of CAD and published in English or in Chinese before May, 2013 were identified by comprehensive computer-based searches of MEDLINE via PubMed (from 1950 to May 2013), EMBASE (1980 to May 2013), the Cochrane Library database (Cochrane Central Register of Controlled Trials, from 1991 to May 2013) and China National Knowledge Infrastructure (CNKI). The following key words were used: “Apolipoprotein M,” “*APOM*,” “rs805296 (T-778C) polymorphism”, “dyslipidemia” and “serum lipid level”, “coronary artery disease”. The above search strategy described was used to obtain titles and abstracts of studies that may have been relevant to this review. The titles and abstracts were screened independently by two authors (Z Zhang and G Chu), who discarded studies that were not applicable. When multiple reports from the same patients were found, only the study with the most complete data set was included in the meta-analysis. But duplicate patients of different articles that have different types of data of outcomes were included both. Any disagreements were arbitrated by discussion with the third reviewer (R-X Yin).

### Included and excluded studies

Two investigators reviewed all identified studies independently to determine whether an individual study was eligible for inclusion. The selection criteria for studies to be considered for this meta-analysis were as follows: (1) case–control studies published in peer-reviewed journals with full available text in English or in Chinese; (2) the APOM gene T-778C polymorphism and serum lipid levels and the risk of CAD; (3) reporting at least one relevant outcomes of association between genotype and serum lipid levels and the risk of CAD, serum lipid levels including TC, HDL-C, LDL-C, and TG. Excluded studies: (1) studies in which it was not possible to extract data from the published results and from the authors; and (2) studies that did not report appropriate outcomes were also excluded.

### Types of outcome measures

(1) Relationship between serum lipid parameters and genotypes; (2) Relationship between the risk of CAD and genotypes; (3) Genotype freguency: TT and CT+CC; (4) Serum lipid parameters: TC, TG, HDL-C, and LDL-C.

### Data extraction and management

Two investigators (Z Zhang and G Chu) independently extracted data according to the author details and the following information was extracted from each study: first author, year of publication, sample size, diagnostic criteria, study design, characteristics of controls, genotype information (number of genotypes, genotyping method, genotype distribution in cases and controls), relationship between genotypes and serum lipid parameters and the risk of CAD. Discrepancies were resolved by discussion. When repeated publications of the same trial were identified, data were extracted from the repeated publications and reported as a single trial.

### Quality assessment

The Newcastle-Ottawa Scale (NOS) [24] was used to assess the quality of each included study of the CAD group. Eight items were contained in the NOS (Table 1). It was categorized into three dimensions including selection, comparability, and exposure for case–control studies. Four, one and three items were contained in the selection, comparability and exposure; respectively. A star system was used to allow a semi-quantitative assessment of the study quality. A study can be awarded a maximum of one star for each numbered item within the selection and exposure categories. A maximum of two stars can be given for comparability. The NOS ranges from zero up to nine stars. High-, medium-, and poor-quality studies should be achieved more than seven, four to six, and less than four stars; respectively.

**Table 1:** Newcastle - Ottawa quality assessment scale case–control studies

**Criteria**	**Score**
**Selection**	
1) Is the case definition adequate?	
a) yes, with independent validation	☆
b) yes, eg record linkage or based on self reports	
c) no description	
2) Representativeness of the cases	
a) consecutive or obviously representative series of cases	☆
b) potential for selection biases or not stated	
3) Selection of controls	
a) community controls	☆
b) hospital controls	
c) no description	
4) Definition of controls	
a) no history of disease (endpoint)	☆
b) no description of source	
**Comparability**	
1) Comparability of cases and controls on the basis of the design or analysis	
a) study controls for the most important factor	☆
b) study controls for any additional factor	☆
**Exposure**	
1) Ascertainment of exposure	
a) secure record (eg surgical records)	☆
b) structured interview where blind to case/control status	☆
c) interview not blinded to case/control status	
d) written self report or medical record only	
e) no description	
2) Same method of ascertainment for cases and controls	
a) yes	☆
b) no	
3) Non-response rate	
a) same rate for both groups	☆
b) non respondents described	
c) rate different and no designation	

### Statistical analysis

Allele frequencies of each study were determined by the allele counting method. Hardy–Weinberg equilibrium (HWE) for each single nucleotide polymorphism was assessed for the controls in each study using Pearson’s Square (*P* > 0.05) [24]. We carried out statistical analysis by the Review Manager software 5.1.0 (updated in March 2011 by the Cochrane Collaboration**)**. We examined the contrast of genetic model (TT *vs*. CT+CC) in serum lipid levels and genetic model (CT+CC *vs*. TT+CT+CC) in the risk of CAD. Continuous variables were expressed as mean difference (MD) with 95% confidence intervals (CI). Dichotomous outcomes of the numbers of CT+CC and TT+CT+CC genotypes in CAD/control values were expressed as odd ratio (OR) with 95% CI. The pooled effects were calculated using fixed effects models when there was no significant heterogeneity but the random effects model was analyzed to ensure robustness of the model chosen and susceptibility to outliers, or using random effects models when there was significant heterogeneity but the fixed effects model was analyzed to ensure robustness of the model chosen and susceptibility to outliers. The point estimate of the MD or OR was considered statistically significant at the 2-tailed *P* ≤ 0.05 level. Heterogeneity was analyzed using a chi-square test on N-1 degrees of freedom [25]. *I*^2^ values of 25%, 50% and 75% correspond to low, medium and high levels of heterogeneity; respectively. Sensitivity analyses were performed omitting a single study at a time or analyzed another model chosen. If enough studies were identified, funnel plots were to be used to investigate reporting biases [26].

## Results

### Characteristics of included studies

**Figure 1:**
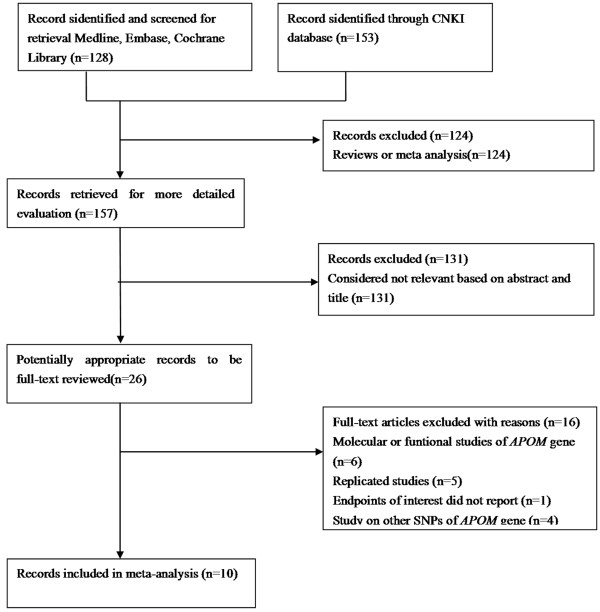
Flow chart showing study selection process.

Ten studies [14-23], 5 in English [15,18,20-22] and 5 in Chinese [14,16,17,19,23] met the included criteria for this meta-analysis (Figure 1). All of the studies had been approved by the Ethics Committee of their affiliations, in accordance with the Helsinki Declaration of 1975 as revised in 1983, and all subjects had given informed consent. The present study was approved by the Ethics Committee of the First Affiliated Hospital, Guangxi Medical University. Among them, six of the eligible studies contained data in lipid parameters on two different groups [14-16,18,21,22], and we treated the different groups as independent. Therefore, a total of 16 separate comparisons were finally included in our meta-analysis.

**Figure 2:**
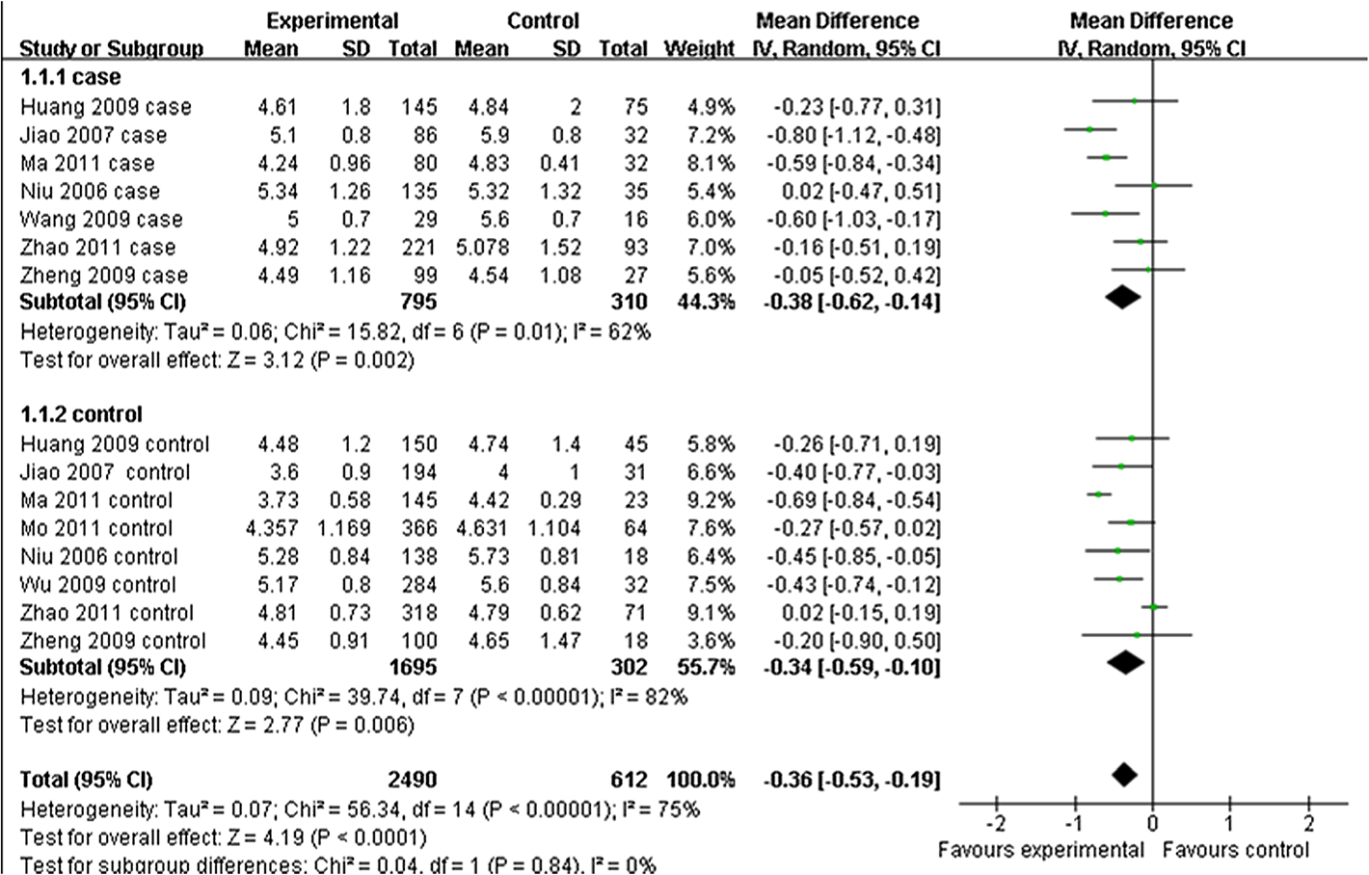
**Forest plot of the association between *APOM *T-778C polymorphism and serum TC levels.** (genetic model: TT *vs*. CT+CC).

**Figure 3:**
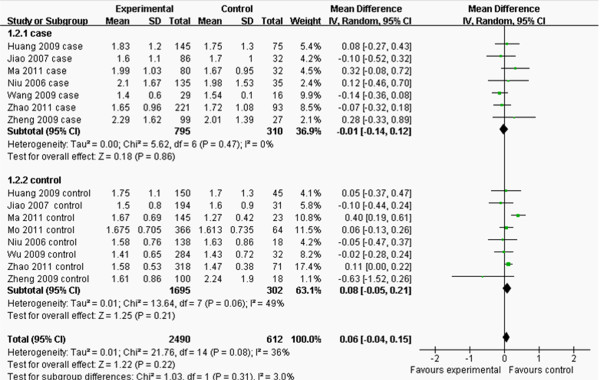
**Forest plot of the association between *APOM *T-778C polymorphism and serum TG levels.** (genetic model: TT *vs*. CT+CC).

**Figure 4:**
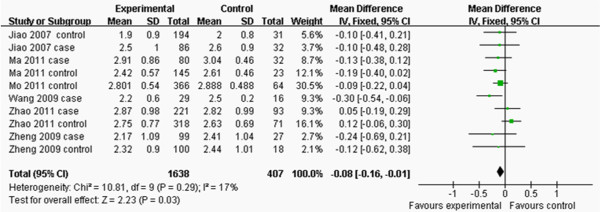
**Forest plot of the association between *APOM *T-778C polymorphism and serum LDL-C levels.** (genetic model: TT *vs*. CT + CC).

**Figure 5:**
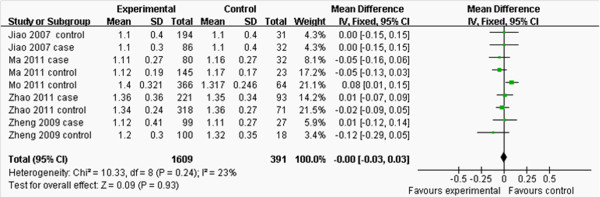
**Forest plot of the association between *APOM *T-778C polymorphism and serum HDL-C levels.** (genetic model: TT *vs*. CT+CC).

**Figure 6:**
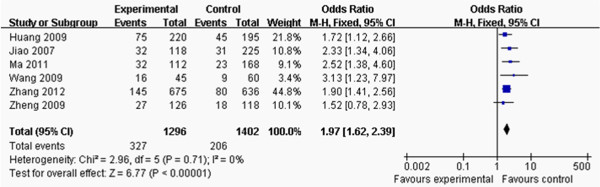
**Forest plot of the association between *APOM *T-778C polymorphism and CAD risk by comparison of CAD and control groups.** (genetic model: CT+CC *vs*. TT+CT+CC).

The main characteristics of each study are shown in Table 2. Nine studies contained the data of TG and TC and there were 15 separated comparisons; six studies contained the levels of LDL-C and there were 10 separated comparisons; five studies included the concentrations of HDL-C and there were 9 separated comparisons; and six studies contained the data of CAD for this meta-analysis (Figures 2, 3, 4, 5 and 6). A total of 4,413 people were included in this study. The relationship between lipid parameters and genotypes in 3,102 people, and between the risk of CAD and genotypes in 2,698 subjects was analyzed; respectively. All the eligible studies were conducted in the Chinese population.

**Table 2:** Baseline characteristics of included studies

**Study**	**Year**	**Eligible subjects (case/control)**	**Age (years)**		**Populations**		**Method**	**Outcome**
			**Case**	**Control**	**Case**	**Control**		
Jiao et al. [15]	2007	118/225	61.8 ± 11.4	60.4 ± 12.7	CAD patients had at least 30% stenos is determined angiographically in at least one of the major segments of coronary arteries, the right coronary artery, left circumflex, or left anterior descending arteries.	Hospital-based	PCR-RFLP	TC,TG, HDL-C, LDL-C
Zhao et al. [21]	2011	314/389	64.43 ± 8.48	63.93 ± 6.86	Ischemic stroke patients included having a sudden loss of global or focal cerebral function, and corresponding infarction confirmed by brain imaging with a computed tomography scan and/or magnetic resonance imaging.	Hospital-based	PCR, SNaPshot	TC,TG, HDL-C, LDL-C
Zheng et al. [22]	2009	126/118	62.5 ±9.4	63.0 ±10.8	Patients that diagnosed as CAD according to the results of angiography (a lesion was classed as being significant when stenosis was >50%).	Hospital-based	real-time PCR	TC,TG, HDL-C, LDL-C, )
Niu et al. [18]	2006	170/156	55.5 ± 13.2	56.2 ± 9.7	T2D patients were diagnosed according to the criteria of the World Health Organization (1999).	Hospital-based	PCR-RFLP	TC, TG
Wu et al. [20]	2009	117/316	22.8 ± 14.5	63.1 ± 9.7	T1D patients were diagnosed according to the criteria of the World Health Organization (1999) with positive of anti-islet antibodies result in inability to produce insulin.	Hospital-based	PCR-RFLP	TC, TG
Mo et al. [17]	2011	-/430	-	-	-	Community-based and hospital staff	PCR-RFLP	TC,TG, HDL-C, LDL-C,
Huang et al. [14]	2009	220/195	64.3 ± 11.1	59.0 ± 10.1	Patients that diagnosed as CAD according to the results of clinical symptoms, angiography, electrocardiogram and echocardiography.	Hospital-based	PCR-RFLP	TC, TG
Wang et al. [19]	2009	45/60	51.4 ± 6.2	52.6 ± 8.6	Patients diagnosed of CAD based on the criteria of the World Health Organization (1979).	Hospital-based	PCR-RFLP	TC,TG, LDL-C
Zhang et al. [23]	2012	675/636	56.4 ± 9.9	55.8 ± 10.4	Patients diagnosed of CAD based on the criteria of the World Health Organization (1979).	Community-based and hospital staff	PCR-RFLP	-
Ma et al. [16]	2011	112/168	57.9 ± 10.2	56.2 ±9.4	Patients that diagnosed as CAD according to the results of angiography (≥50% stenos in at least one of the major segments of coronary arteries).	Hospital-based	Allele-specific PCR	TC,TG, HDL-C, LDL-C

*CAD* Coronary artery disease, *T2D* Type 2 diabetes mellitus, *T1D* Type 1 diabetes mellitus, *PCR* Polymerase chain reaction, *RFLP* Restriction fragment length polymorphism, *TC* Total cholesterol, *TG* Triglycerides, *HDL-C* High-density lipoprotein cholesterol, *LDL-C* Low-density lipoprotein cholesterol.

The quality of all included studies was assessed by NOS (Table 3). Most studies were of medium or high quality in terms of selection and exposure. However, as for comparability, the quality was relativity low, since some of confounders didn’t be adjusted for the analysis.

**Table 3:** Quality assessment for all the included studies of the CAD

**First author**	**Publishing year**	**Selection**	**Comparability**	**Exposure**
Jiao [15]	2007	☆☆☆	☆☆	☆☆☆
Huang [14]	2009	☆☆☆	-	☆☆☆
Zheng [22]	2009	☆☆☆	☆	☆☆☆
Ma [16]	2011	☆☆	☆	☆☆
Zhang [23]	2012	☆☆☆☆	-	☆☆
Wang [19]	2009	☆☆☆	-	☆☆

### Frequencies of the genotype

Frequencies of the *APOM* T-778C genotypes in each group and all *P*-values of HWE testing are shown in Table 4. The genotype distribution of the *APOM* T-778C polymorphism in all eligible studies was consistent with HWE (*P* > 0.05).

**Table 4:** **Genotypic frequency of *APOM *** **T-778C polymorphisms in different populations included in the meta-analysis**

**Study**	**Year**	**Case (%)**			**Control (%)**			**P-Value for Hardy-Weinberg equilibrium**
		**TT**	**CT**	**CC**	**TT**	**CT**	**TT**	
Jiao et al. [15]	2007	86 (72.9)	29 (24.6)	3 (2.5)	194 (86.2)	31 (13.8)	0 (0.0)	0.267
Zhao et al. [21]	2011	221 (70.4)	86 (27.4)	7 (2.2)	318 (81.7)	67 (17.3)	4 (1.0)	0.823
Zheng et al. [22]	2009	99 (78.6)	25 (19.8)	2 (1.6)	100 (84.7)	18 (15.3)	0 (0.0)	0.370
Niu et al. [18]	2006	135 (79.4)	34 (20.0)	1 (0.6)	138 (88.5)	18 (11.5)	0 (0.0)	0.444
Wu et al. [20]	2009	140 (79.1)	37 (20.9)	0 (0.0)	284 (89.9)	32 (10.1)	0 (0.0)	0.358
Mo et al. [17]	2011	-	-	-	366 (85.1)	62 (14.4)	2 (0.05)	0.717
Huang et al. [14]	2009	145 (65.9)	66 (30.0)	9 (0.41)	150 (76.9)	41 (21.0)	4 (2.1)	0.548
Wang et al. [19]	2009	29 (64.4)	15 (33.3)	1 (2.2)	51 (85.0)	9 (15.0)	0 (0.0)	0.530
Zhang et al. [23]	2012	530 (78.5)	135 (20.0)	10 (1.5)	556 (87.5)	74 (11.6)	6 (0.9)	0.052
Ma et al. [16]	2011	80 (71.4)	30 (26.8)	2 (1.8)	145 (86.3)	21 (12.5)	2 (1.2)	0.231

### Outcomes of lipid levels

As shown in Figure 2, the pooled effects indicated that CT+CC group had higher levels of TC than TT group (MD:-0.36, 95% CI: -0.53 – -0.19, *P* < 0.0001; heterogeneity test *x*^*2*^ = 56.34, *P* < 0.00001, *I*^2^ = 75%). There was no difference in the levels of TG between TT and CT+CC groups (MD: 0.06, 95% CI: -0.04–0.15, *P* = 0.22; heterogeneity test *x*^*2*^ = 21.76, *P* = 0.08, *I*^*2*^ = 36%; Figure 3). CT+CC group had higher levels of LDL-C than TT group (MD: -0.08, 95% CI: -0.16– -0.01, *P* = 0.03; heterogeneity test *x*^*2*^ = 10.81, *P* =0.29, *I*^*2*^ = 17%; Figure 4). There was no difference in the levels of HDL-C between TT and CT+CC groups (MD: 0.00, 95% CI: -0.03–0.03, *P* = 0.93; heterogeneity test *x*^*2*^ = 10.33, *P* = 0.24, *I*^*2*^ = 23%; Figure 5).

### O**utcomes of the risk of CAD**

The eligible compared groups were pooled with fixed effects models and the data of CT+CC and CC+CT+TT groups in the CAD/control value and the pooled effects are shown in Figure 6. Pooled effects showed that CAD group had higher CT+CC genetype frequency than control group (OR: 1.97, 95% CI: 1.62–2.39, *P* < 0.00001; heterogeneity test *x*^*2*^ = 2.96, *P* = 0.71, *I*^*2*^ = 0%).

### Sensitivity analysis

Sensitivity analyses were performed to assess the contribution of each study to the pooled estimate and by excluding individual studies one at a time and recalculating the pooled MD or OR estimates for the remaining studies. Eliminating the studies with more than 300 patients or less than 100 patients in each group did not substantially change the pooled point estimate, which indicates the reliability of our results. What is more, performing transition of model also did not substantially change the pooled point estimate.

### Heterogeneity analysis

For the outcomes of TC, the *I*^2^ value of heterogeneity was greater than 50% and the heterogeneity test: *P* value was less than 0.10 in model TT *vs*. CT+CC in the overall populations, which indicated statistically significant heterogeneity among the studies. In order to explore the sources of heterogeneity, the studies were stratified by cases and controls and subgroup analyses were performed. However, heterogeneity still existed in the outcomes of TC (Figure 2).

### Publication bias

**Figure 7:**
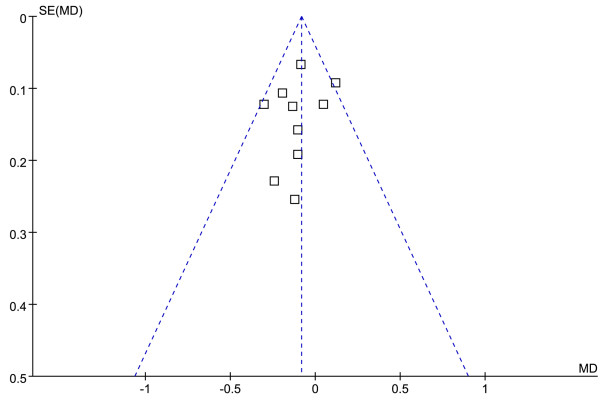
**Funnel plot of the association between *APOM *T-778C polymorphism and serum LDL-C levels.** (genetic model: TT *vs*. CT + CC).

**Figure 8:**
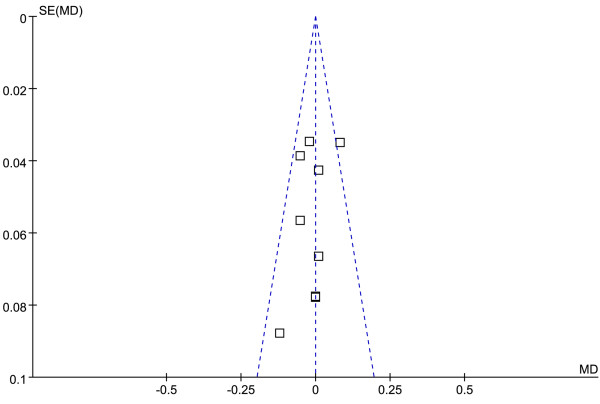
**Funnel plot of the association between *APOM *T-778C polymorphism and serum HDL-C levels.** (genetic model: TT *vs*. CT + CC).

The funnel plot and Egger test were performed to assess the publication bias of literatures in all comparison models. No visual publication bias was found in the funnel plot (Figures 7 and 8).

## Discussion

Association of different genes in relation to the predisposition of CAD has been widely examined. It may conclude that CAD is both multifactorial and polygenic in nature. The disorders of lipid profile are important risk factors involved in this process [27]. A variety of candidate genes have been investigated as predisposing factors of CAD, including those involved in lipid metabolisms. ApoM is one of the latest additions to the apolipoprotein family [28]. Over expression of ApoM had a protective effect against atherosclerosis [12]. Many investigators carried out case–control studies in order to determine the association between this polymorphism and serum lipid levels or the risk of CAD, but the results are inconclusive. This may be due to a small sample size. The characteristic of a meta-analysis is to obtain a more competitive result by combining the comparable studies, increasing the sample size and statistical power [29].

The results of our meta-analysis showed that the CT+CC group had higher levels of TC and LDL-C than the TT group, and there was no difference in the levels of HDL-C and TG between the TT and CT+CC groups. Moreover, the CT+CC genotype was associated with increased risk of CAD.

In the present study, we included ten studies and all of the subjects were the Chinese populations. Baseline characteristics of two comparison groups of included studies were substantially similar. All included studies were consistent with HWE. The quality of the included studies that associated between polymorphism and CAD risk was assessed by NOS and most studies were of medium or high quality. In addition, heterogeneities of pooled effects of HDL-C, LDL-C and CAD were low. MD of total of TC ( *P* < 0.0001) and OR of total of CAD risk (*P* < 0.00001) were large effect. All these made our conclusion more robust.

Heterogeneity analysis showed that the polymorphism suggested significant heterogeneity in the outcomes of TC in the overall populations. To explore the sources of heterogeneity, we performed subgroup analyses, but heterogeneity still existed in the outcomes of TC. This may be due to China being a large country with multiethnic country. Different groups of the included studies had different genetic background and environmental factors. It is well known that both serum lipid levels and CAD are affected by genetic and environmental factors, such as dietary patterns, lifestyle, obesity, physical inactivity. Moreover, pharmaceuticals, age, gender, hypertension are also associated with serum lipid levels [30].

Some limitations of this meta-analysis should be discussed. First, this meta-analysis focused only on papers published in English and Chinese and the ones that reported in other languages may bias the present results. Second, the meta-analysis cannot be corrected the bias of each study in the outcomes of TC, and may generate a statistical conclusion with more power and precision. In addition, gene-gene and gene-environment interactions should also be considered in the analysis.

Therefore, it is necessary to conduct large sample studies using standardized unbiased genotyping methods, homogeneous CAD patients and well- matched controls. Moreover, gene-gene and gene-environment interactions should also be considered in the analysis. Such studies taking these factors into account may eventually lead to better, more comprehensive understanding of the association between the *APOM* T-778C polymorphism and serum lipid levels or the risk of CAD.

## Conclusion

The results of the current meta-analysis showed that the CT+CC group had higher levels of TC and LDL than the TT group, and there was no difference in the levels of HDL and TG between the TT and CT+CC groups. Moreover, there a prominent association between the *APOM* T-778C polymorphism and the risk of CAD in the Chinese population, and the CT+CC genotype was associated with increased risk of CAD.

## Competing interests

The authors declare that they have no competing interests.

## Authors’ contributions

ZZ and GC conceived the study, participated in the design, collected the data, performed statistical analyses, and drafted the manuscript. RXY conceived the study, participated in the design, and helped to draft the manuscript. All authors read and approved the final manuscript.
